# Supplementary material for: Local Genomic Surveillance of Invasive Streptococcus pyogenes in Eastern North Carolina (ENC) in 2022–2023
Source: Int J Mol Sci. 2024 Jul 26;25(15):8179. doi: 10.3390/ijms25158179 (PMC11311789; doi:10.3390/ijms25158179)
Supplement: Supplementary file 1 [file ijms-25-08179-s001.zip › SuppFigs.pptx]

## Slide 1
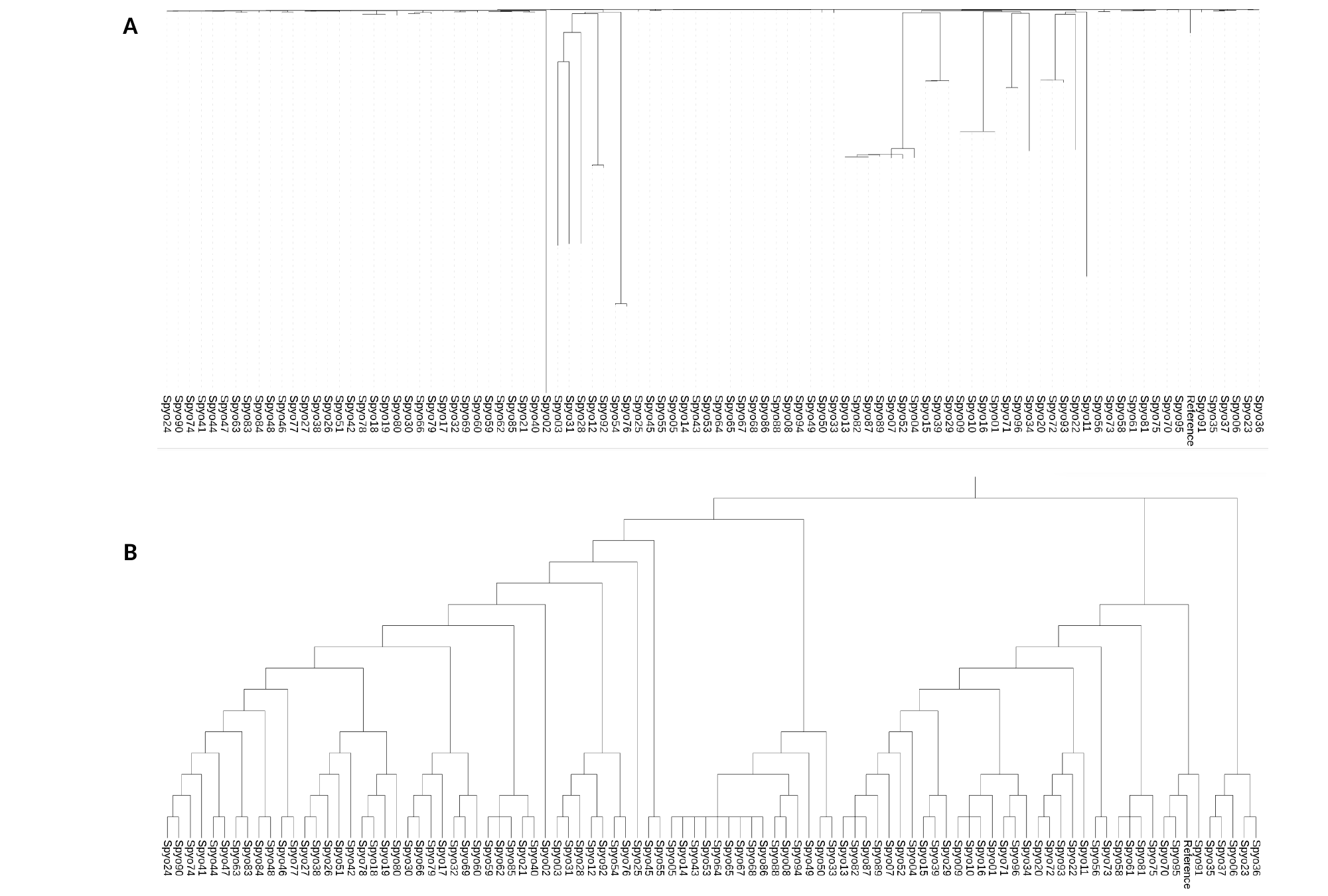

A
B

## Slide 2
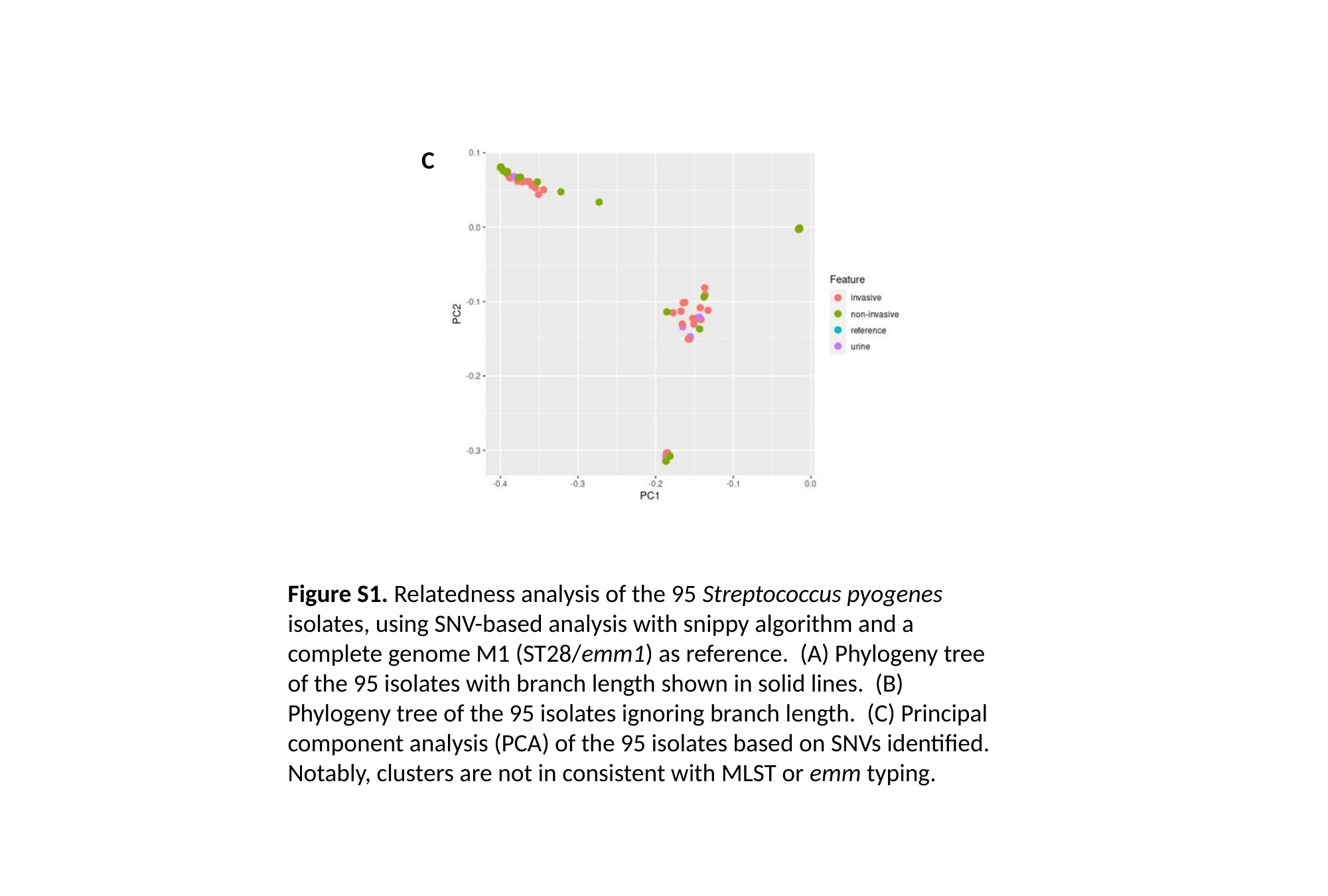

C
Figure S1. Relatedness analysis of the 95 Streptococcus pyogenes isolates, using SNV-based analysis with snippy algorithm and a complete genome M1 (ST28/emm1) as reference. (A) Phylogeny tree of the 95 isolates with branch length shown in solid lines. (B) Phylogeny tree of the 95 isolates ignoring branch length. (C) Principal component analysis (PCA) of the 95 isolates based on SNVs identified. Notably, clusters are not in consistent with MLST or emm typing.

## Slide 3
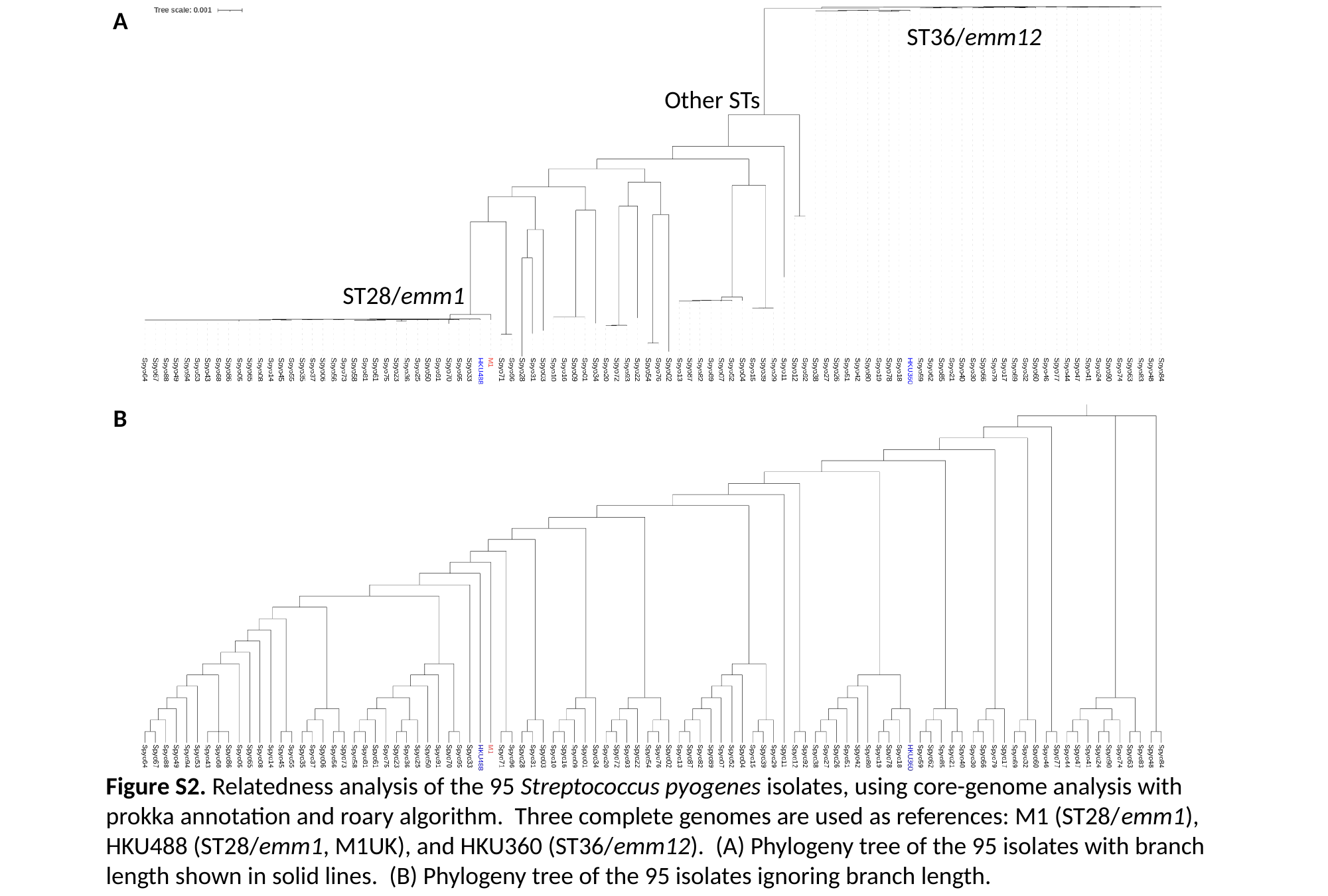

A
ST36/emm12
Other STs
ST28/emm1
B
Figure S2. Relatedness analysis of the 95 Streptococcus pyogenes isolates, using core-genome analysis with prokka annotation and roary algorithm. Three complete genomes are used as references: M1 (ST28/emm1), HKU488 (ST28/emm1, M1UK), and HKU360 (ST36/emm12). (A) Phylogeny tree of the 95 isolates with branch length shown in solid lines. (B) Phylogeny tree of the 95 isolates ignoring branch length.

## Slide 4
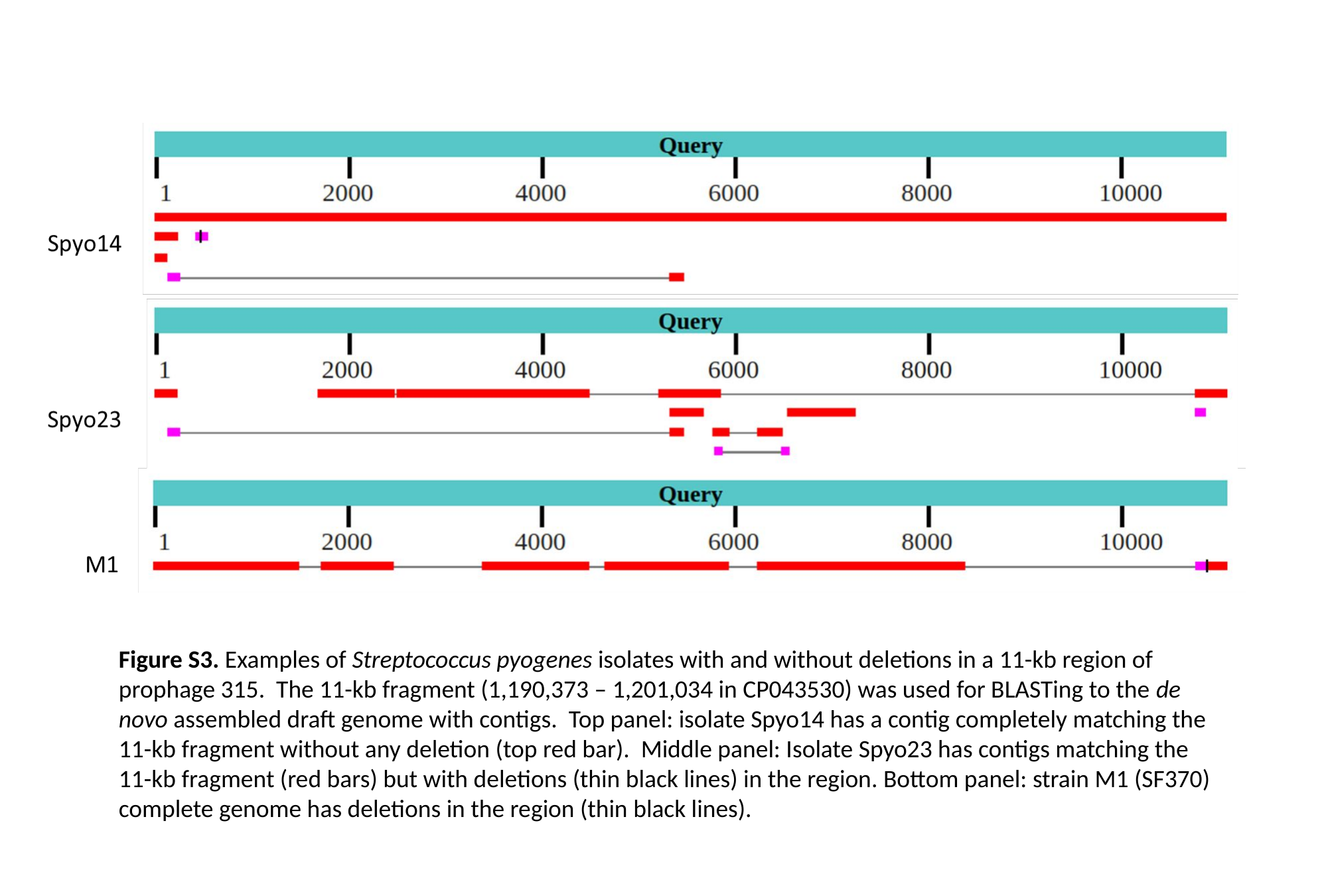

Figure S3. Examples of Streptococcus pyogenes isolates with and without deletions in a 11-kb region of prophage 315. The 11-kb fragment (1,190,373 – 1,201,034 in CP043530) was used for BLASTing to the de novo assembled draft genome with contigs. Top panel: isolate Spyo14 has a contig completely matching the 11-kb fragment without any deletion (top red bar). Middle panel: Isolate Spyo23 has contigs matching the 11-kb fragment (red bars) but with deletions (thin black lines) in the region. Bottom panel: strain M1 (SF370) complete genome has deletions in the region (thin black lines).

## Slide 5
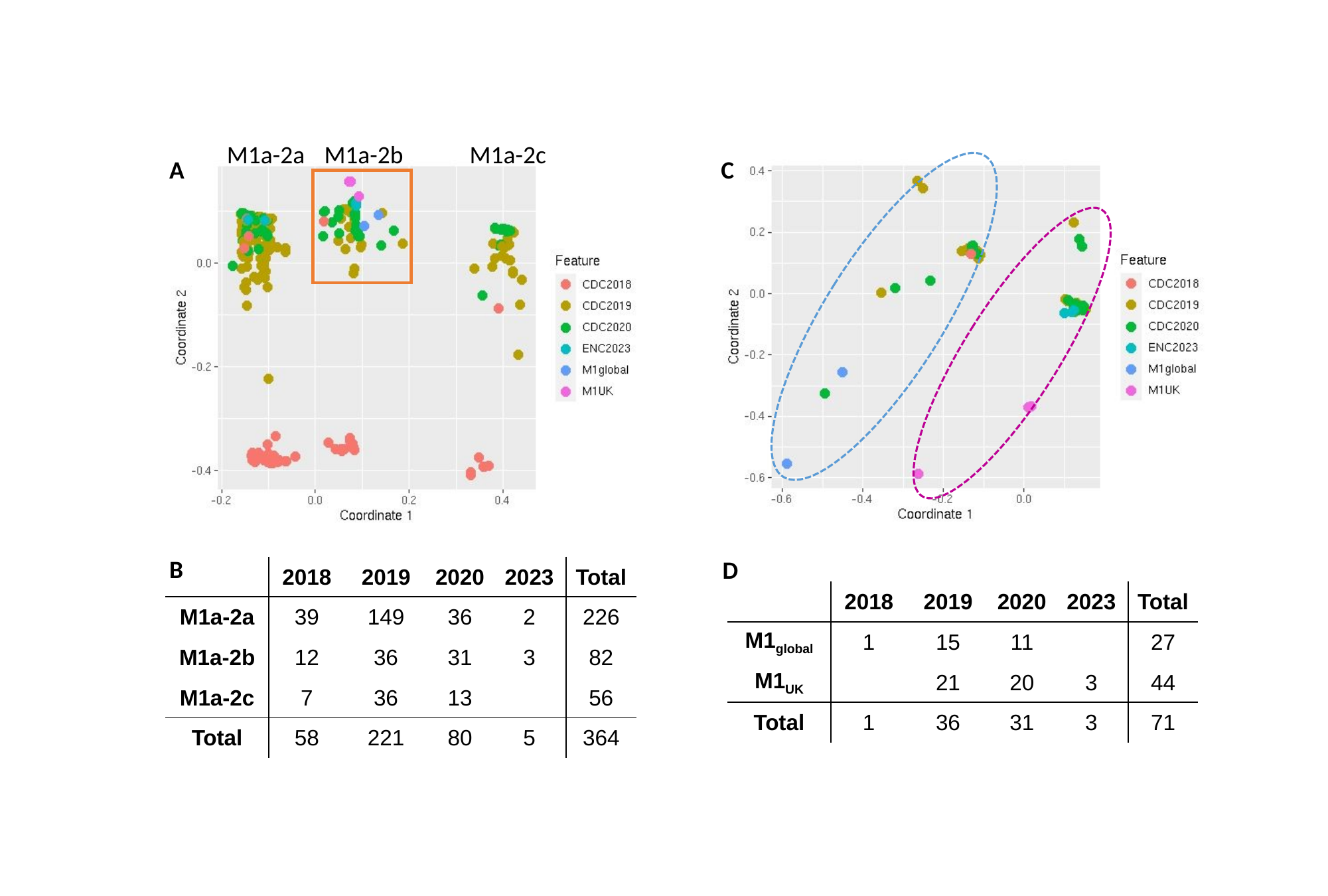

M1a-2a
M1a-2b
M1a-2c
A
C
B
D
| | 2018 | 2019 | 2020 | 2023 | Total |
| --- | --- | --- | --- | --- | --- |
| M1a-2a | 39 | 149 | 36 | 2 | 226 |
| M1a-2b | 12 | 36 | 31 | 3 | 82 |
| M1a-2c | 7 | 36 | 13 | | 56 |
| Total | 58 | 221 | 80 | 5 | 364 |
| | 2018 | 2019 | 2020 | 2023 | Total |
| --- | --- | --- | --- | --- | --- |
| M1global | 1 | 15 | 11 | | 27 |
| M1UK | | 21 | 20 | 3 | 44 |
| Total | 1 | 36 | 31 | 3 | 71 |

## Slide 6
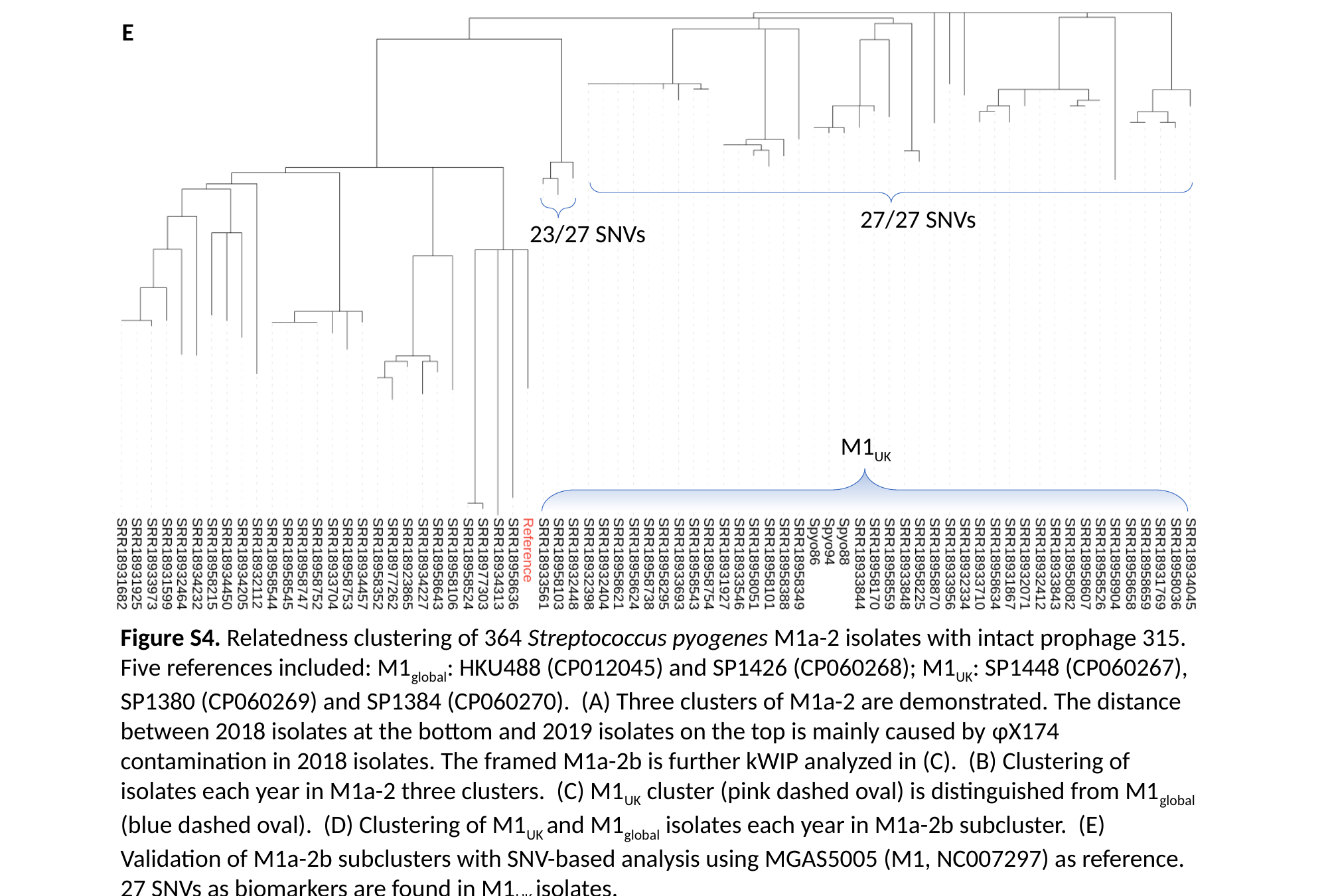

E
27/27 SNVs
23/27 SNVs
M1UK
Figure S4. Relatedness clustering of 364 Streptococcus pyogenes M1a-2 isolates with intact prophage 315. Five references included: M1global: HKU488 (CP012045) and SP1426 (CP060268); M1UK: SP1448 (CP060267), SP1380 (CP060269) and SP1384 (CP060270). (A) Three clusters of M1a-2 are demonstrated. The distance between 2018 isolates at the bottom and 2019 isolates on the top is mainly caused by φX174 contamination in 2018 isolates. The framed M1a-2b is further kWIP analyzed in (C). (B) Clustering of isolates each year in M1a-2 three clusters. (C) M1UK cluster (pink dashed oval) is distinguished from M1global (blue dashed oval). (D) Clustering of M1UK and M1global isolates each year in M1a-2b subcluster. (E) Validation of M1a-2b subclusters with SNV-based analysis using MGAS5005 (M1, NC007297) as reference. 27 SNVs as biomarkers are found in M1UK isolates.
